# Removal of Stains and Spots

**Published:** 1880-12

**Authors:** 


### REMOVAL OF STAINS AND SPOTS.

*Matter Adhering Mechanically.*—Beating, brushing, and currents of water either on the upper or under side.

*Gum, Sugar, Jelly, etc.*—Simple washing with water at a hand heat.

*Grease.*—White goods, wash with soap or alkaline lyes. Colored cottons, wash with lukewarm soap lyes. Colored woolens the same, or ammonia. Silks, absorb with French chalk or fuller's earth, and dissolve away with benzine or ether.

*Oil Colors, Varnish and Resins.*—On white or colored linens, cottons or woolens, use rectified oil of turpentine, alcohol, lye and then soap. On silks, use benzine, ether, and mild soap, very cautiously.

*Stearine.*—In all cases, strong pure alcohol.

*Vegetable Colors, Fruit, Red Wine, and Red Ink.*—On white goods, sulphur fumes or chlorine water. Colored cottons and woollens, wash with lukewarm soap, lye or ammonia. Silk the same, but more cautiously.

*Alizarine Inks.*—White goods, tartaric acid, the more concentrated the older are the spots. On colored cottons and woollens, and on silks, dilute tartaric acid is applied, cautiously.

*Blood and Albuminoid Matters.*—Steeping in lukewarm water. If pepsine, or the juice of *Carica papaya*, can be procured, the spots are first softened with lukewarm water, and then either of these substances is applied.

*Iron Spots and Black Ink.*—White goods, hot oxalic acid, dilute muriatic acid, with little fragments of tin. On fast dyed cottons and woollens, citric acid is cautiously and repeatedly applied. Silks, impossible.

*Lime and Alkalies.*—White goods, simple washing. Colored cottons, woolens, and silks are moistened, and very dilute citric acid is applied with the finger end.

*Acids, Vinegar, Sour Wine, Must, Sour Fruits.*—White goods, simple washing, followed up by chlorine water if a fruit color accompanies the acid. Colored cottons, woolens, and silks are very carefully moistened with dilute ammonia, with the finger end. [In case of delicate colors, it will be found preferable to make some prepared chalk into a thin paste, with water, and apply it to the spots.]

*Tanning from Chestnuts, Green Walnuts, etc., or Leather.*—White goods, hot chlorine water, and concentrated tartaric acid.

Colored cottons, woolens, and silks, apply dilute chlorine water cautiously to the spot, washing it away and reapplying it several times.

*Tar, Cart Wheel Grease, Mixtures of Fat, Resin, Carbon, and Acetic Acid.*—On white goods, soap and oil of turpentine, alternating with streams of water. Colored cottons and woolens, rub in with lard, let lie, soap, let lie again, and treat alternately with oil of turpentine and water. Silks the same, more carefully, using benzine instead of oil of turpentine.

*Scorching.*—White goods, rub well with linen rags dipped in chlorine water. Colored cottons, redye if possible, or in woolens raise a fresh surface. Silks, no remedy.

